# Poor adherence to guidelines for preventing central line-associated bloodstream infections (CLABSI): results of a worldwide survey

**DOI:** 10.1186/s13756-016-0139-y

**Published:** 2016-11-22

**Authors:** Cristina Valencia, Naïma Hammami, Antonella Agodi, Alain Lepape, Eduardo Palencia Herrejon, Stijn Blot, Jean-Louis Vincent, Marie-Laurence Lambert

**Affiliations:** 1 Healthcare Associate Infections Unit, Scientific Institute of Public Health, Brussels, Belgium; 2 European Programme for Interventional Epidemiology Training (EPIET), ECDC, Stockholm, Sweden; 3 Department GF Ingrassia, University of Catania, Catania, Italy; 4 Department Anaesthesia, General Intensive Care, University hospital, Lyon, France; 5 Intensive Care department, Hospital Universitario “Infanta Leonor”, Madrid, Spain; 6 Department of Internal Medicine, Ghent University, Ghent, Belgium; 7 Department of Intensive Care, Erasme University, Brussels, Belgium

**Keywords:** Healthcare associated infection, Central line-associated bloodstream infections, Bloodstream infections, Preventive practices, Intensive care units, Surveillance

## Abstract

**Background:**

Central line-associated bloodstream infections (CLABSI) are a cause of increased morbidity and mortality, and are largely preventable. We documented attitudes and practices in intensive care units (ICUs) in 2015 in order to assess compliance with CLABSI prevention guidelines.

**Methods:**

Between June and October 2015, an online questionnaire was made available to medical doctors and nurses working in ICUs worldwide. We investigated practices related to central line (CL) insertion, maintenance and measurement of CLABSI-related data following the SHEA guidelines as a standard. We computed weighted estimates for high, middle and low-income countries using country population as a weight. Only countries providing at least 10 complete responses were included in these estimates.

**Results:**

Ninety five countries provided 3407 individual responses; no low income, 14 middle income (MIC) and 27 high income (HIC) countries provided 10 or more responses. Of the total respondents, 80% (MIC, SE = 1.5) and 81% (HIC, SE = 1.0) reported availability of written clinical guidelines for CLABSI prevention in their ICU; 23% (MIC,SE = 1.7) and 62% (HIC,SE = 1.4) reported compliance to the following (combined) recommendations for CL insertion: hand hygiene, full barrier precaution, chlorhexidine >0.5%, no topic or systemic antimicrobial prophylaxis; 60% (MIC,SE = 2.0) and 73% (HIC,SE = 1.2) reported daily assessment for the need of a central line. Most considered CLABSI measurement key to quality improvement, however few were able to report their CLABSI rate. Heterogeneity between countries was high and country specific results are made available.

**Conclusions:**

This study has identified areas for improvement in CLABSI prevention practices linked to CL insertion and maintenance. Priorities for intervention differ between countries.

**Electronic supplementary material:**

The online version of this article (doi:10.1186/s13756-016-0139-y) contains supplementary material, which is available to authorized users.

## Background

Central line-associated bloodstream infections (CLABSI) occurring in intensive care units (ICUs) are a cause of increased morbidity and mortality, and are largely preventable [1–3]. CLABSI prevention requires evidence based clinical practices on the one hand and monitoring of these clinical interventions on the other hand.

Clinical practices can be classified in three categories, (1) practices at central line (CL) insertion (such as maximal barrier precautions, avoiding femoral vein if possible); (2) practices during maintenance of a CL; and (3) reduction of exposure to a CL (such as daily assessment of the need for CL and timely removal of unnecessary CL) [4, 5]. Several studies in high income countries have shown that adequate use of CLABSI prevention measures can significantly reduce CLABSI rate [6–9].

Studies have also shown that elementary infection control measures may reduce the incidence of CLABSIs in low and middle income countries significantly, amounting to a reduction from 6.5 to 46.0 cases per 1000 CL days to 2.4–12.4 cases per 1000 CL days [10–15]. Yet, CLABSI rates reported in low and middle income countries, where resources are limited, are much higher than CLABSI rates reported in high income countries [16].

Although evidence-based prevention practices for CLABSI have been established, ensuring sustained adherence to them is a challenge [16, 17]. For this reason, measurement of outcomes and processes is an essential component of any intervention aimed at improving quality of care [5].

This study aimed to document, attitudes and practices (clinical and measurement) regarding CLABSI prevention in ICUs in low, middle and high income countries in order to assess compliance with CLABSI prevention guidelines, its measurement and identify priorities for interventions.

## Methods

### Study setting and population

Our study population comprised of medical doctors and nurses working in an ICU in the year 2015. The medical doctor and nurse could work in the same ICU and more than one reply was possible from one unit.

An ICU was defined as a unit meeting all of the following criteria: provides facilities for invasive mechanical ventilation, and pump-controlled administration of infusion, functions 24 h a day and 7 days a week, and there is at least one doctor immediately available at all times to deal with emergencies.

### Questionnaire

An online questionnaire was developed and consisted of five parts: 1) Characteristics of the respondent and ICU setting, 2) Clinical practices for CL insertion, 3) Clinical practices for CL maintenance, 4) Monitoring of outcomes and processes and 5) Attitudes towards measurement as a tool for improvement. We used as a reference clinical practice guidelines published by the Society for Healthcare Epidemiology of America (SHEA) [4]. We included questions on the measurement of outcomes (CLABSI rate and the ability to report selected indicators) and measurement of processes such as compliance with prevention practices, including hand hygiene, and the device utilization ratio (ratio of central-line days to patient-days). Attitudes regarding the implementation of a data collection system was measured using a 5 point-Likert scale (1: strongly agree, 5: strongly disagree) [17, 18].

The questionnaire was developed and pretested in English and translated into 9 languages (Spanish, German, Portuguese, Italian, French, Dutch, Russian, Mandarin and Japanese). Native speaker intensive care doctors and/or infection control practitioners translated the questionnaire. Each translation was independently verified by a second native speaking physician. Participation was anonymous.

We used Limesurvey ® 2.0, an open source web survey application, to collect the data [19].

### Dissemination to target group

The questionnaire was available online from 10 June 2015 to 31 October 2015. It was endorsed by 5 international societies (the European Society of Intensive Care Medicine (ESICM), the Society of Critical Care Medicine (SCCM), the World Federation of Societies of Intensive Care and Critical Medicine (WFSICCM), the International Symposium on Intensive Care and Emergency Medicine (ISICEM) and the Middle East Critical Care Assembly (MCCA)), and one national society (Japanese society of intensive care medicine (JSCIM)). Endorsement implied mailing to members; and/or posting on the website. We also identified and contacted national ICU societies who advertised the questionnaire on their website and pertaining congresses, and developed mass mailings to all its members.

### Sample size

No sample size or power calculations were conducted.

### Data analysis

Descriptive statistics were used to summarize characteristics of the study population Standard errors were calculated by dividing the standard deviations of each estimate by the square root of the sample size (n).

Based on the 2015 World Bank classification [20] we categorized countries as low, middle and high- income. We computed weighted estimates for middle and high-income countries using total country population [20] as the weight (to correct for contributing responses from each country) for those that provided at least 10 completed replies (arbitrary cut-off).

Analyses were conducted using STATA 13 (svyset and svy commands for survey data for weighted estimates).

Detailed country specific data are available as a Additional file 1. Data are freely available and have been deposited in the Dryad Digital Repository: http://dx.doi.org/10.5061/dryad.f7h12

Further use and exploitation of these data is encouraged.

## Results

Three thousand four hundred seven complete individual responses were received from 95 countries. Weighted estimates and standard errors for 14 middle and 27 high income countries are based on 3,250 responses received from the 41 countries from which at least 10 completed replies were available. No low-income countries provided 10 or more responses. Out of 3407 respondents, 157 (4%) came from 55 countries for which less than 10 replies were given; 2414 (71%) came from high income countries with at least 10 replies and 836 (25%) came from 14 middle income countries with at least 10 replies. The distribution per country is given in Table 1.

**Table 1:** Number of respondents to the survey by country, 2015

	Replies^a^	%^b^
High income countries		
United States	401	17
Belgium	226	9
Spain	207	9
Russia	199	8
France	183	8
Japan	175	7
United Kingdom	135	6
Switzerland	120	5
Australia	110	5
Canada	103	4
Italy	82	3
Germany	62	3
Qatar	56	2
Portugal	53	2
Denmark	42	2
New Zealand	37	2
Argentina	37	1
Saudi Arabia	29	1
Netherlands	28	1
Norway	28	1
Greece	20	1
Poland	20	1
Sweden	15	1
Venezuela	12	0.5
Chile	14	0.5
Uruguay	10	0.5
Austria	10	0.5
*Total responses of 27 high income countries with at least 10 replies*	2414	100
Middle income countries		
China	379	45
India	130	16
Brazil	92	11
Jordan	38	5
Colombia	38	5
Mexico	24	3
Costa Rica	20	2
Lebanon	19	2
Sudan	19	2
South Africa	18	2
Turkey	18	2
Peru	16	2
Ecuador	13	2
Ukraine	12	1
*Total responses of 14 Middle Income countries with at least 10 replies*	836	100

^a^Replies : total number of respondents per country providing a complete questionnaire

^b^%: the proportion of total respondents per country

The characteristics of the respondents and their setting by income country are presented in Table 2. 40% of the respondents were nurses and 60% doctors. Of those who reported availability of written guidelines for CLABSI prevention, approximately 80% of the respondents in both middle and high income countries reported that the guidelines were last revised within the last 5 years.

**Table 2:** Characteristics of the setting and respondent by income category in 2015

	Middle income countries (14 countries)		High income countries (27 countries)	
	*N* = 836	SE*	*N* = 2,414	SE*
Setting				
Admissions per year in their ICU (mean)	1,024	59	1,123	36
N beds in ICU (mean)	22	0.8	19	0.5
Type of Hospital (University) (%)	65	2.0	59	1.5
Written guidelines for CLABSI prevention (answer yes) (%)	80	1.5	81	1.0
These guidelines include.... (%)				
Indications for central-line	70	1.9	56	1.4
Process of insertion for central-line	71	1.9	72	1.2
Maintenance of central-line	75	1.8	75	1.1
Indications for removal of central-line	66	2.0	52	1.5
Hand hygiene done using alcohol based hand rubs (always/most of the times)	92	1.9	94	1.5
Respondent				
Years working in ICU (mean)	8	0.3	16	0.3
Gender (females) (%)	55	2	44	1.5
Profession (doctors) (%)	59	2	70	1.4

Absolute numbers are not reported because percentages are weighted estimates

*Standard error

CLABSI prevention clinical practices as reported by ICU doctors and nurses are presented in Table 3. In middle income countries, the use of chlorhexidine >0.5% for skin preparation and full body drape during CL insertion were less commonly implemented. Overall, only 23% and 62% of respondents from middle and high income countries reported full compliance to the recommended practices and avoided antimicrobial prophylaxis.

**Table 3:** CLABSI prevention: clinical practices during insertion and maintenance by income category, as reported by ICU doctors and nurses in 2015

	Middle income countries (14 countries)		High income countries (27 countries)	
	*N* = 836		*N* = 2,414	
	%*	SE**	%*	SE**
Procedures at insertion				
*In my ICU, the following procedures were used during the last catheter insertion I did or assisted in…*				
Hand hygiene before central line insertion	97	0.7	98	0.3
Using mask, cap, sterile gloves and sterile gown	93	1.0	96	0.5
Using chlorhexidine >0.5% in alcohol for skin preparation	58	2.1	85	0.9
Using sterile drapes to cover patient from head to toes	51	2.1	82	0.9
Using antimicrobial ointment at insertion site (NR***)	14	1.4	9	0.9
Administering systemic antimicrobial prophylaxis for CLABSI prevention (NR***)	24	1.8	8	0.7
Four recommended practices and none of not recommended	23	1.7	62	1.4
Insertion site				
*Ultrasound guidance was used last time central line was inserted in the internal jugular vein* (%yes)	50	2.1	79	1.1
In my ICU, the femoral vein is used always, or most of the time (NR***)	15	1.3	11	0.9
Procedures at maintenance				
*When I last accessed a central-line, I…*				
Disinfected central line hubs when accessing ports	84	1.5	82	1.0
Disinfected my hands before	87	1.4	88	0.9
Used gloves	59	2.1	84	1.4
*Change of dressing (if not soiled, damped or loose)*				
Dry Dressing (sterile gauze)				
every 2 days	20	1.7	15	1.0
more often (NR***)	31	1.9	24	1.2
less often (NR***)	34	2.0	29	1.1
Don’t know	15	1.5	32	1.4
Transparent Dressing				
every 5-7 days	22	1.7	41	1.5
more often (NR***)	70	1.9	40	1.2
less often (NR***)	2	0.5	2	0.4
Don’t know	6	0.9	17	1.1
*In my ICU, patients with a central line are assessed daily for the need or removal of it (explicitly: with a note in clinical file, or a tick on a check list)*	60	2.0	73	1.2

*Absolute numbers are not reported because percentages are weighted estimates

**Standard error

***Not recommended

Measurements on compliance with CLABSI prevention guidelines reported by ICU doctors and nurses are presented in Table 4. The majority of respondents in middle and high income countries say they monitor regularly CLABSI rates, but only a minority can actually report those rates. The monitoring of process indicators is lower in high compared to middle income countries.

**Table 4:** CLABSI prevention: measurements by income category, as reported by ICU doctors and nurses in 2015

Measurements	Middle income countries (14 countries)		High income countries (27 countries)	
	*N* = 836		*N* = 2414	
	%^a^	SE^b^	%^a^	SE^b^
*Measurement of compliance to recommendations at least one a year*				
Central line insertion	72	2.0	66	1.5
Daily assessment of a need for a central line	72	2.0	59	1.5
Disinfection practices when accessing the central line	73	2.0	59	1.5
Hand hygiene	81	2.1	73	1.5
*“In my ICU, there is a written definition of CLABSI for data collection”*				
Yes	78	1.7	70	1.2
*“In my ICU, we count and record, routinely…” (% saying “yes”)*				
CLABSI	73	1.8	81	0.9
Days since last CLABSI	39	2.0	57	1.4
*Respondents reporting data (%)- for part or all of 2015*				
CLABSI Rate (either per central-line days, or per patient-days)	16	1.5	26	1.4
*“Clinical staff in my ICU is aware of CLABSI-related data, and their trends” (% agree strongly/agree)*	76	2.0	67	1.4

^a^Absolute numbers are not reported because percentages are weighted estimates

^b^Standard Error

Attitudes of doctors and nurses towards the implementation of a measurement system of infection in ICUs are presented in Table 5. The majority of respondents in middle and high income countries have a positive attitude towards measurement to stimulate quality improvement.

**Table 5:** Attitudes towards the implementation of a measurement system of infections in ICUs by income category, as reported by ICU doctors and nurses in 2015

	Middle income countries (14 countries)				High income countries (27 countries)			
	*N* = 836				*N* = 2414			
	Agree strongly/agree		Disagree/disagree strongly		Agree strongly/agree		Disagree/disagree strongly	
	%^a^	SE^b^	%^a^	SE^b^	%^a^	SE^b^	%^a^	SE^b^
*To what extent do you agree with the following comments*								
If you cannot measure it, you cannot improve it	81	2.0	3	0.7	80	1.5	9	0.8
Monitoring of CLABSI related measures stimulates quality improvement	96	2.0	1	0.3	91	1.5	1	0.7
These data can be used against me	21	1.0	55	1.0	24	0.8	52	1.4
CLABSI-related data in my ICU (if any) are reliable	74	2.0	2	0.2	68	1.5	6	0.6
I am willing to implement, or support, a CLABSI data collection system	92	2.0	1	0.1	88	1.5	1	0.1
Clinical diagnosis of CLABSI is difficult: this makes measurement systems unreliable	40	1.9	24	1.0	27	1.2	44	1.4
There is a difference between a definition of CLABSI for reporting, and a diagnosis of CLABSI for treatment	54	2.1	12	0.4	47	1.4	23	1.1

^a^Absolute numbers are not reported because percentages are weighted estimates

^b^Standard Error

Wide variations exist between countries. Less than half of the respondents report availability of guidelines in their ICU in Japan, (78/145, 45%) or Russia (90/199, 45%), but nearly all do in the US (383/401, 96%). In France (35/183) and China (72/379) one in five respondents (19%) report that femoral vein is used “always, or most of the time” for CL insertion. In Belgium, 34% (76/226) report daily assessment of need of CL while in the UK this was 90% (121/135). Compliance with all four recommended practices without the use of the 2 not-recommended during CL insertion was high in Spain (176/207, 85%) and the US (323/401, 81%), but low in Belgium (72/226, 32%), France (41/183, 22%), Japan (52/175, 30%) and Russia (46/199, 23%). Complete data can be found in the Additional file 1.

## Discussion

### Key results

This study represents, to our knowledge, the first international survey assessing CLABSI prevention practices (clinical and measurement), and attitudes towards measurement reported by ICU doctors and nurses. More than 3,000 provided complete responses, 40% of them were nurses.

A majority of respondents (80%) report the existence of CLABSI prevention guidelines in their ICU, demonstrating a wide awareness of the CLABSI problem. However several areas for improvement have been identified. Combined compliance to 4 recommended practices without the use of the 2 not-recommended practices (such as antimicrobial prophylaxis) at CL insertion was low particularly in middle income countries, the weakest point being in the utilization of sterile drape to cover patients from head-to-toe and the use of >0.5% chlorhexidine for skin preparation. Only a minority of respondents still report the not recommended femoral vein as the most used insertion site in their ICU. During maintenance, dressings are changed more often than recommended and assessment of need of the CL is not always done on a daily basis. Despite most of the respondents agreeing that measurement is essential for improvement, and routinely counting CLABSI, only few were able to report the actual CLABSI rate in their unit. Our findings are comparable to several other studies or surveys conducted on CLABSI prevention practices in ICU’s at local level [21–23]. A survey assessing implementation of preventive CLABSI measures and monitoring of compliance to these measures, identified that the use of full body drape during CVC insertion was among the measures less applied. Moreover, less than 50% of hospitals included in this study reported monitoring compliance to recommended measures [22]. Finally, Furuya et al. identified that daily line checks and optimal site selection were least commonly implemented. In this study only a disappointing 38% of those that monitored bundle implementation reported full compliance, this aligns with our low reported numbers. The availability of a bundle policy and monitoring of compliance and a compliance of at least 95% were needed to decrease CLABSI rates [23].

### Strengths and limitations

The strengths of this study are the large number of respondents, the proportion of nurses (40%) and the ability to provide country-specific results for a large number of countries. However, our results are based on a non-random sample of respondents, we have not been able to compute a response rate, and we do not know the number of ICUs as the online questionnaire did not include questions allowing for the identification of the ICU, in order to preserve the anonymity of the respondents**.** In addition, given the dissemination strategy-through international and national societies-some categories of ICU doctors and nurses are likely to be overrepresented. Members of these societies might be better informed, and might have better prevention practices, than non-respondents to the survey. Those working in ICUs where CLABSI prevention guidelines exist might have been more motivated to participate in this survey. Reported practices are likely to differ from actual practices and be biased towards what the respondent believed to be desirable. These selection and reporting biases would lead to our results overestimating true compliance with recommended practices.

We used SHEA guidelines, but these are not “global” standards and some prevention measures recommended in SHEA guidelines might be controversial such as disinfection with chlorhexidine 0.5% in specific settings [24]. However it was beyond the scope of this article to assess the evidence supporting each recommendation. Detailed information for each recommendation is given in the country-specific file, so that the usefulness of this information can be assessed against national or local guidelines.

The grouping of our data in order to provide estimates for “high income” and “middle income” countries was a convenient way to aggregate the data, but given the heterogeneity of practices between countries these summary estimates have limited meaning, and the most relevant data can be found in the country-specific results presented in the Additional file 1.

We did not explore whether results differed between nurses and doctors (this will be the subject of a subsequent research).

### Interpretation

The large number of respondents, and the large proportion of ICUs where CLABSI prevention guidelines are available, likely reflect the interest and awareness of the ICU community in the issue of CLABSI prevention. Selection and reporting bias in our study lead to the overestimation of CLABSI prevention practices in ICU’s, therefore weaknesses identified seem robust enough to identify interventions for improvement. Priorities for improvement differ between countries; from encouraging the use of chlorhexidine >0.5% in alcohol for skin preparation and sterile drapes to covering patients from head to toes, to discouraging the use of the femoral vein as preferred insertion site. Improving knowledge of clinical guidelines is far from sufficient to improve practices but it is a prerequisite [25].

Availability and discussion of ICU data is also very important as it can serve to highlight opportunities and areas for improvement. The majority of respondents agree that monitoring of CLABSI-related measures stimulates quality improvement but very few actually knew their data. In order for clinical staff to monitor trends over time and report real time feed-back they should be educated in how to generate reliable data through indicators that measure compliance. A compromise needs to be found between time-consuming data collection, and usefulness of data. Simple measures promoted by hospital management can be implemented to increase knowledge of CLABSI data at ICU level, e.g. a panel with the number of days since last ICU-acquired infection (including CLABSI) can be displayed on the wall and updated daily.

## Conclusions

This international study shows that there is clear interest and awareness in the ICU community for CLABSI prevention in high and middle income countries, but implementation and adherence to existing guidelines on insertion and maintenance of CL need to be reinforced at ICU level. Areas for improvement in practices (clinical and measurement) related to CLABSI prevention in ICUs have been identified and include: full barrier precautions, reduction of device exposure through daily assessment of CL, and utilization of data to monitor progress in preventive actions. Priorities for improvement differ from country to country. It would be advantageous to continue to address factors that may be affecting the adoption and consistent use of CLABSI prevention guidelines, as well as encouraging collaboration on ICU accountability by close monitoring of infection rates, giving feedback to staff, and establishing a reliable data collection system.

## Additional file

Additional file 1: Country Specific Results _xls. (XLS 214 kb)

## Abbreviations

CHX: Chlorhexidine
CL: Central line
CLABSI: Central line-associated bloodstream infection
CVC: Central venous catheter
ECDC: European center for disease prevention and control
EPIET: European programme for intervention epidemiology training
ESICM: European Society of Intensive Care Medicine
HIC: High income country
ICU: Intensive care unit
ISICEM: International Symposium on Intensive Care and Emergency Medicine
JSCIM: Japanese society of intensive care medicine
MCCA: Middle East Critical Care Assembly
MIC: Middle income country
SCCM: Society of Critical Care Medicine
SE: Standard errors
SHEA: Society of Healthcare Epidemiology of America
WFSICCM: World Federation of Societies of Intensive Care and Critical Medicine
